# Gender Differences in Work and Well-Being in Later Life

**DOI:** 10.1177/01640275251353217

**Published:** 2025-06-21

**Authors:** Juryung Kaitlyn Cho

**Affiliations:** 1 Department of Social Policy and Intervention, 6396 University of Oxford, Oxford, UK

**Keywords:** educational level, extended working life, gender, SES, subjective well-being, South Korea

## Abstract

This study examines the longitudinal relationship between work status and subjective well-being (SWB) among older adults in South Korea, with a focus on the roles of gender and education. Using data from the Korean Longitudinal Study of Aging (2006–2020), this study employs fixed-effects regression models to examine within-person longitudinal associations between work and SWB, stratified by gender and educational level. Findings reveal a positive association between working status and SWB for men, but not for women. Both lower- and higher-educated individuals experience a positive SWB when they are working. While women in South Korea are more likely to have lower educational attainment, this does not fully explain the gender gap in the relationship between work status and SWB. Policies should address barriers preventing women from experiencing similar SWB benefits as men, ensuring work supports, rather than strains, their well-being.

## Introduction

Subjective well-being (SWB), a concept introduced by Diener (1984), is widely studied in the social sciences, and commonly assessed through measures of life satisfaction (Ramia & Voicu, 2020). Life satisfaction captures an individual’s subjective evaluation of their overall quality of life, encompassing the presence of positive experiences and emotions, the absence of negative ones, and a holistic assessment of life as a whole rather than momentary feelings (OECD, 2024; Stutzer & Frey, 2010). SWB in South Korea (henceforth, Korea) is notably low compared to other industrialised countries, particularly among older adults. Koreans rated their life satisfaction at an average of 5.8 out of 10, which is below the Organisation for Economic Co-operation and Development (OECD) average of 6.7, ranking Korea 35th out of 41 countries according to this metric (OECD, 2024). This is an actual cause for concern, as negative SWB is strongly associated with suicide risk (Wu et al., 2014), while conversely, improving SWB can help reduce suicidal ideation (Liu et al., 2024). Alarmingly, older adults in Korea are the source of one of the highest suicide rates among OECD countries, covering the period of 2003–2019 (Jang et al., 2022), reflecting serious challenges to their SWB.

Korea also has the highest labor force participation rate among older adults and the highest old-age poverty rate among OECD countries (OECD, 2020). This paradox highlights that, despite ranking highest in the employment component of the Active Aging Index (Um et al., 2019), many older Koreans may remain in the workforce due to financial necessity rather than personal choice (Kim et al., 2018). Together with Mexico, Korea is the only country where the share of wage income for older people exceeds 50% of that total income (compared to the OECD average of 25.8%) (OECD, 2021). This is primarily due to the late introduction of a national pension system in 1988, which provided limited coverage and inadequate benefits. Many older adults are financially vulnerable, as fewer than 50% received a pension in 2015 (Kim et al., 2018).

In the Korean context, work status is closely tied to financial security in later life. In a broader international context, interest in later-life employment and its implications for SWB has grown, particularly as welfare retrenchment compels older adults to remain in the labor force for longer (Sohier, 2019), and pension systems increasingly fail to cover the rising costs of long-term care and medical needs (Ebbinghaus & Möhring, 2022). Despite this heightened attention, research on the relationship between work status and SWB in later life remains limited. Studies suggest that the intersection of extended working lives and growing economic insecurity produces complex and deeply gendered experiences (Léime & Ogg, 2019). Educational attainment remains a crucial factor, as early-life disadvantages such as limited schooling constrain access to secure and well-paying employment throughout the life course (Di Gessa et al., 2018). These dynamics underscore the importance of examining how gender and education moderate the link between work status and SWB. Nevertheless, such considerations are often overlooked in prevailing discussions on employment in later life.

This study examines the longitudinal relationship between work status and SWB, with a focus on the moderating roles of gender and education. It contributes to research on aging, work, and well-being by investigating how later-life employment is experienced differently across these dimensions. Rather than assuming that work in later life is uniformly beneficial, this research contributes to a more nuanced understanding by raising questions about dominant narratives that promote extended working lives and by highlighting the need for policy approaches that account for heterogeneities among older people in the context of where employment is often a necessity rather than a choice.

## Theoretical Framework

To understand the relationship between work status and well-being in later life, this article draws from key concepts of activity theory (Havighurst, 1961), role theory (Goode, 1960; Sieber, 1974), conservation of resources theory (Hobfoll, 1989), and cumulative disadvantage theory (O’Rand, 1996). The latter three perspectives question whether the activity theory’s claim about the positive impact of work status on well-being applies equally across different gender and educational backgrounds. As a result, the moderating effects of gender and education on the relationship between work status and well-being emerge as key areas for further investigation.

### Work Status and SWB in Later Life

Activity theory (Havighurst, 1961), which has played a pivotal role in shaping the active aging framework since the 1990s, posits that continued participation in work-related activities enhances the well-being of older adults by fostering activity and social engagement. Work status is also deeply tied to holding defined roles and accessing resources in later life. In addition to being a source of self-conception, employment provides individuals with social connections, support, and opportunities for rewards (Reitzes & Mutran, 1994).

Conservation of resource theory (Hobfoll, 1989) suggests that employment acts as a critical source of financial resources, which are integral to well-being in later life (Litwin & Sapir, 2009). For older adults, the loss of employment through retirement often leads to a decline in income, which can create financial insecurity. This insecurity has far-reaching effects, increasing stress, anxiety, and depression while negatively impacting physical health, mental well-being, and overall quality of life (Olivera & Ponomarenko, 2017). The financial stability gained through continued employment or sufficient retirement benefits not only supports basic needs but also enables participation in social and leisure activities, further enhancing their overall sense of well-being. In South Korea, the inadequacy of pension and welfare benefits leaves many older adults in poverty, with earned income serving as their primary source of financial support in later life.

While the literature on well-being in later life presents varied perspectives, the importance of earning income to sustain livelihoods for many older adults in Korea suggests that employment is generally associated with greater SWB than unemployment. This leads to the formulation of the following baseline hypothesis: *Working status will be positively linked to SWB in later life (H1)*. The following sections examine how gender and educational level may influence this relationship, informing the development of more specific hypotheses.

### Work Status, SWB, and Gender

Role theory lends itself to two contradictory formulations, which can be summarized as role enhancement and role strain hypotheses. They suggest that men and women experience work status differently due to societal expectations and gendered roles (Eagly, 1987). From the role enhancement perspective, holding multiple *meaningful* roles, such as employment, positively influences well-being in later life by fulfilling personal needs (Rozario et al., 2004; Sieber, 1974). Conversely, the role strain perspective argues that employment may increase overload and conflict, reducing SWB, particularly when individuals face competing demands from multiple roles (Goode, 1960; Marks, 1977). Traditional gender roles and gender-specific occupational experiences significantly influence engagement in productive activities, with notable differences in the prevalence and perceived importance of paid versus unpaid work (Plaisier et al., 2008). These differences highlight the need for gender-specific analyses to better understand their actual impacts over time.

Empirical studies also demonstrate that the association can differ among men and women due to role identity and multiple roles. Older men tend to define their identity largely in terms of their employment status, making the worker role a crucial aspect of their psychological well-being (Quick & Moen, 1998). As a result, involuntary or unexpected job loss in later life – whether via unemployment or retirement – poses a significant challenge, with research showing more pronounced negative effects on male well-being compared to that perceived by women (Unger et al., 2018). By contrast, the literature suggests that older women experience increased stress as they navigate the dual demands of work, caregiving, and household responsibilities, which can negatively impact their well-being (DePasquale et al., 2016; Eagly, 1987). By contrast, Ashwin and her colleagues (2021) found that employment provided a noneconomic SWB boost for Russian older female pensioners. In interviews, they found that even with low-status work, female pensioners were more likely to achieve social recognition while working than during full retirement, and employment improved the status in the households. In contrast to these mixed findings, Sohier (2019) found no difference between men and women, concluding that employment benefits both equally, with no observed interaction effect.

In the Korean context, older men are more likely to have been socialized into worker and breadwinner roles, making employment central to their identity and sense of purpose. In contrast, women are primarily socialized for caregiving responsibilities throughout their lives (Lee & Yeung, 2021). Consequently, continued employment is expected to be associated with role enhancement for older men but is more likely linked to role strain for older women. Given the significant role of gender in shaping work experiences, this leads to the hypothesis that *the positive association between working status and SWB will be more pronounced among older men than older women (H2)*.

### Work Status, SWB, and Education Levels

Understanding how disparities in financial resources are shaped by educational attainment is crucial. Cumulative disadvantage theory emphasizes the persistent effects of early disadvantages, such as low educational attainment, which limit access to well-paying jobs and career advancement opportunities later in life (Crystal & Shea, 1990; O’Rand, 1996). In line with cumulative disadvantage theory, limited access to training and educational opportunities for low-income workers further exacerbates these disparities, hindering their ability to secure higher-paying positions as they age (Zhan & Pandey, 2004). People with limited educational attainment often work in physically demanding jobs with limited resources and autonomy, while those with higher education levels tend to occupy roles offering more control, skill use, and well-being (Taylor & Geldhauser, 2007).

Conservation of resource theory (Hobfoll, 1989) complements cumulative disadvantage theory by providing insight into how educational differences can moderate the relationship between work status and well-being in later life. From this perspective, cumulative disadvantage can lead to resource depletion, as individuals in lower-status jobs have fewer opportunities to develop and maintain key resources, such as financial security, job autonomy, and social capital. Given the importance of financial resources for well-being, those in lower-status jobs, often linked to lower education, are expected to experience lower well-being in later life than those in higher-status jobs with greater resources.

Empirical findings consistent with these theories suggest that working beyond state pension age enhances well-being when it is a voluntary choice, but diminishes it when driven by financial necessity, highlighting the roles of resources and autonomy in shaping later-life work outcomes (Di Gessa et al., 2018; Nikolova & Graham, 2014). Similarly, Siegrist et al. (2017) found that lower well-being among older workers is closely linked to poor job quality, reinforcing the importance of working conditions in determining the benefits of later-life employment. However, other studies suggest a more uniform well-being benefit of later-life employment. Sohier (2019) found that employment status is positively associated with well-being, with no statistically significant interaction between the total years spent in full-time education and employment. Similarly, research using panel data from the UK and Germany has shown that post-retirement employment generally improves SWB, regardless of occupational class (Lux & Scherger, 2017).

Although the findings are mixed, in the Korean context, where employment-related financial resources are critical for later-life well-being, higher-educated older adults are expected to benefit more from work that provides psychological fulfillment and financial security, while those with lower education may gain fewer well-being benefits from holding lower-status jobs with limited financial rewards. Based on these perspectives, the hypothesis presented in this section is that *the positive relationship between working status and SWB will be more pronounced among older adults with higher levels of education than those with lower levels of education (H3)*.

## Methods

### Data

This study uses data from the Korean Longitudinal Study of Aging (KLoSA), a nationally representative panel survey conducted every two years. KLoSA provides detailed information on individuals’ socio-demographic characteristics, health, family relationships, employment, and intergenerational financial support. The baseline sample in 2006 consisted of 10,254 individuals, selected through a stratified, multistage sampling method. To address non-response and attrition, the survey applies design, non-response, and post-stratification weights, maintaining representativeness over time. This study draws on data from Waves 1 to 8, focusing on individuals aged 50 to 80 to capture later-life employment transitions while limiting bias from frailty and attrition at older ages. In Korea, exiting one’s main job in midlife is often a temporary transition rather than a permanent retirement, as many re-enter the workforce and continue working into later life (Boo & Chang, 2006).

### Measures

#### Dependent Variable

This study uses SWB as the dependent variable, measured through life satisfaction, a widely recognised indicator of SWB in later life. Life satisfaction reflects an individual’s overall evaluation of their life across various dimensions and aspirations as it provides a relatively stable cognitive assessment of general well-being (Diener et al., 2018; George, 2010). In this study, life satisfaction is assessed with the question: “On a scale of 0–100, where zero means completely dissatisfied and 100 means completely satisfied, how satisfied are you with your life?” It is treated as a continuous interval variable ranging from 0 to 100, with higher values indicating greater satisfaction.

#### Independent Variable

The main explanatory variable is a binary indicator of work status, which identifies whether an older adult reports being employed or self-employed versus not participating in the labor force at the time of the interview. Individuals who continue working during their retirement years are categorized as “employed,” even if they already receive a pension. In Korea, many older adults work beyond the mandatory retirement age of 60. This trend reflects the instability of midlife employment and the typical pattern of early retirement from primary (career) jobs, followed by re-employment —often in low-quality, non-standard jobs or self-employment, rather than continued work with the same employer— resulting in prolonged participation in the labor market (Boo & Chang, 2006).

#### Covariates

The present study incorporated a series of control variables previously identified in the literature as pertinent predictors of the SWB of older people. I coded a set of control variables, including self-rated health status, marital status, living arrangements, and place of residence, to address potential confounding factors and improve the comprehension of the association between the main variables of interest.

Firstly, a potential association exists between an individual’s self-rated health and capacity to engage in work-related activities. Self-rated health refers to an individual’s evaluation of their overall health condition. It is indicative of their subjective assessment of their physical and mental state. There is a potential correlation between low self-rated health and impairments in both physical and cognitive capabilities, which can impact an individual’s capacity to participate in employment and subsequently affect their overall well-being (Kenny et al., 2008). I thus included a time-varying indicator for self-rated health on a 5-point scale ranging from 1)very poor, 2)poor, 3)good, 4)very good, 5)excellent. Activities of daily living (ADL) limitations were included to account for physical health constraints that could affect individuals’ ability to work and well-being (Boccaccio et al., 2021). In addition, being married (or in a committed relationship) may offer individuals emotional support, companionship, and a feeling of inclusion, potentially enhancing their overall well-being (Carr et al., 2014). Economic resources can also be linked to marital status; for example, married people may have access to joint financial resources (earnings and/or pensions), which could provide greater financial security and well-being. Similarly, family changes and the number of people living in a household are also substantially associated with SWB (Dolan et al., 2008). Marital status is coded as a binary variable (not married, married). I also included a categorical indicator of household size (living alone, living with one person, living with more than one person), as living alone in later life is widely associated with a negative impact on SWB (Jonestone et al., 2021). Depressed mood over the past week was included to capture short-term mental health fluctuations that can independently affect life satisfaction (Van Damme-Ostapowicz, 2021). Also caring for a family member with ADL limitations was included to account for caregiving responsibilities, which may limit labor market participation and impact well-being (Qu & Burr, 2024). SWB of individuals can also be influenced by their place of residence, which can be attributed to a range of environmental factors. Urban areas have the potential to offer increased accessibility to a wide range of amenities, services, and recreational facilities, which can positively impact an individual’s overall SWB. It has also been indicated that older adults in rural areas are prone to having lower educational attainment, encountering increased poverty rates, demonstrating inferior health, and confronting higher mortality rates in comparison to their urban peers (Bane & Bull, 2001). The region was coded as 1) big cities, 2) small and mid-sized cities, and 3) rural areas.

#### Analytical Strategy

This study employs a panel analysis to examine the relationship between labor market involvement and SWB. Combining cross-sectional and time-series data enables this approach to account for temporal dynamics while controlling for unobserved, time-invariant individual characteristics. A fixed-effects (FE) model is used to estimate the association between employment status and well-being, as it effectively handles unobserved heterogeneity across individuals, such as personality traits or cultural influences (Bell & Jones, 2015). Unlike pooled ordinary least squares, the FE model isolates the effects of time-varying variables by focusing on within-individual variation, providing robust and reliable estimates. The Hausman test confirmed the appropriateness of the FE model over the random-effects approach (*p* = .000) for all genders, indicating that FE is the best fit for this analysis. The model accounts for time-varying variables, such as work status, while controlling for broader trends and confounders. This is particularly important as personality traits, which explain a significant portion of variance in SWB (Sohier, 2019), are time-invariant and often overlooked in other approaches. Because time-constant variables such as gender and education levels cannot be directly analyzed in an FE model, separate panel regression models were constructed for subgroups stratified by gender and education levels. This reflects the expectation that employment patterns and their effects on SWB differ by these factors.

## Results

Drawing on data from eight waves of KLoSA (2006–2020), Table 1 presents the baseline characteristics of men and women aged 50 to 80 for the first wave. The employment rate (for the age group 50–80) was about 52.6% for men and 21.2% for women in 2006 (wave 1), while across all waves (2006–2020), in terms of all observations, 55.7% of men and 29.2% of women were in working status, suggesting an increase over time particularly for women. Women also had lower levels of educational attainment, with only 21.4% of working women and 17.4% of non-working women having completed high school or higher, compared to 50.9% of working men and 41.4% of non-working men. This disparity likely reflects historical inequalities in access to education, shaped by social norms, economic barriers, and limited opportunities for women in earlier generations. Additionally, a higher proportion of working men (50.9%) and women (21.4%) had completed high school or higher, compared to their non-working counterparts (41.4% for men and 17.4% for women).

**Table 1.** Descriptive Statistics at Wave 1 (2006), Korea Longitudinal Study of Aging.

Variables	Non-Working Men	Working Men	Non-Working Women	Working Women
Life satisfaction				
Mean	58.115	66.275	58.434	61.467
SD	22.754	18.342	22.793	19.986
	Prop. (%)	Prop. (%)	Prop. (%)	Prop. (%)
Education level				
Elementary school or lower	42.1	29.0	67.7	60.3
Middle school	16.5	20.1	14.9	18.3
High school or higher	41.4	50.9	17.4	21.4
Marital status				
Not married	10.3	4.5	35.6	25.8
Married	89.7	95.5	64.4	74.2
Self-reported health				
Very poor	10.8	1.2	9.5	4.4
Poor	27.3	11.4	34.8	26.0
Fair	32.8	31.6	32	32.5
Very good	26.7	50.7	21.9	34.7
Excellent	2.4	5.1	1.8	2.4
Region of residence				
Big cities	45.4	42.5	46.5	37.7
Small and mid-sized cities	33.9	30.3	32.3	27.2
Towns and villages	20.7	27.2	21.2	35.1
Living arrangements				
Living alone	4.7	2.5	15.1	11.7
Living with one person	55.1	40.2	41.0	42.8
Living with more than one	40.2	57.3	43.9	45.5
ADLs				
0	92	99.4	95.2	99.6
1	1.5	0.4	1.8	0.3
2	1.1	0.1	0.7	0
3+	5.4	0.1	2.3	0.1
Felt depressed over the past week				
Felt that way briefly or did not feel that way at all	70.9	87.0	65.7	74.3
Sometime felt that way (for a day or two)	20.2	10.4	23	20.0
Often felt that way (for about 3-4 days)	6.5	2.0	8.8	4.9
Always felt that way (for about 5–7 days)	2.4	0.6	2.5	0.8
Relationship quality with spouse				
Low	21.1	12.8	26.1	23.7
High	78.9	87.2	73.9	76.3
Relationship quality with children				
Low	21.8	13	20.3	16.1
High	78.2	87	79.7	83.9
Care for a family member with ADL difficulties				
Does not provide care	97.2	98.0	96.7	96.8
Provides care	2.8	2	3.3	3.2
Number of observations	1655	1839	3468	934

Examining self-rated health, working individuals of both genders were more likely to report fair or better health. In contrast, non-working men and women showed a higher prevalence of poor or very poor health. Notably, a higher proportion of older working women reported poor or very poor health compared to working men, indicating potential gendered health disparities within the workforce. Health status emerges as a salient differentiator between working and non-working older adults, consistent with expectations around age-related health decline and labor force participation. Notably, only 26.7% of non-working men and 21.9% of non-working women reported very good health, compared to 50.7% and 34.7% of their working counterparts, respectively, while reports of very poor health were substantially higher among the non-working (10.8% for men; 9.5% for women) than among the working (1.2% for men; 4.4% for women). Mental health patterns showed similar patterns, with 2.4% of non-working men and 2.5% of non-working women reporting feeling depressed for 5–7 days in the past week, compared to just 0.6% and 0.8% among their working counterparts. Caregiving responsibilities showed limited variation across groups, with approximately 2–3% of individuals in each category reporting care for a family member with ADL limitations.

Table 2 presents estimates of the association between work status and SWB using FE models. Model 1, analysing the total sample, examines the association between work status and well-being among older adults. Models 2 and 3 explore potential gender differences in this association, while Models 4 and 5 assess potential differences by educational level. The stratified analyses reveal a consistent positive association between work status and SWB, although this relationship is not statistically significant for older women. There is a positive link between work status and SWB among both higher- and lower- educational levels, while it is stronger among older adults with higher education levels than those with lower education.

**Table 2.** FE Results Using Total Sample and Stratified Data by Gender and Education Levels, With Life Satisfaction as a Dependent Variable.

Model	(1)	(2)	(3)	(4)	(5)
	SWB	SWB	SWB	SWB	SWB
Sample	Total	Men	Women	Lower edu (Middle school or lower)	Higher edu (High school or higher)
Estimation model	Fixed effects				
Work status (ref: Not working)					
Working	1.511*** (0.335)	2.947*** (0.506)	0.398 (0.447)	1.420*** (0.465)	1.596*** (0.479)
Self-rated health status (ref: Excellent)					
Very good	−1.963*** (0.670)	−0.861 (0.921)	−3.291*** (0.981)	−2.252** (1.131)	−1.742** (0.807)
Good	−3.399*** (0.681)	−2.267** (0.944)	−4.730*** (0.988)	−3.843*** (1.126)	−3.038*** (0.837)
Fair	−5.959*** (0.724)	−4.845*** (1.024)	−7.207*** (1.036)	−6.542*** (1.158)	−5.230***
Poor	−9.031*** (0.954)	−8.794*** (1.418)	−9.710*** (1.310)	−9.395*** (1.370)	−8.238*** (1.586)
ADL limitations (ref: 0)					
1	−1.803 (1.342)	−2.984 (1.927)	−0.530 (1.880)	−0.248 (1.600)	−7.160*** (2.627)
2	−0.915 (2.364)	0.202 (3.062)	−2.343 (3.765)	−2.701 (2.816)	4.961 (4.585)
3+	−7.915*** (1.268)	−6.091*** (1.781)	−9.714*** (1.836)	−8.465*** (1.496)	−6.941*** (2.584)
Depressed mood over the past week (ref: Felt that way briefly or did not feel that way at all)					
Sometimes felt that way (for a day or two)	−2.077*** (0.259)	−2.175*** (0.411)	−1.988*** (0.333)	−1.835*** (0.342)	−2.461*** (0.398)
Often felt that way (for about 3-4 days)	−3.359*** (0.477)	−3.479*** (0.773)	−3.289*** (0.606)	−3.344*** (0.583)	−3.300*** (0.872)
Always felt that way (for about 5–7 days)	−4.504*** (0.917)	−3.117** (1.461)	−5.366*** (1.175)	−6.098*** (1.140)	−1.582 (1.593)
Marital status (ref: Married)					
Not married	−11.56 (11.07)	−14.94 (14.88)	−8.869 (16.67)	−7.681 (17.51)	−14.35 (13.93)
Relationship quality with spouse (ref: Low)					
High	7.646*** (0.308)	6.774*** (0.510)	8.114*** (0.385)	7.150*** (0.394)	8.516*** (0.502)
Relationship quality with children (ref: Low)					
High	9.239*** (0.350)	8.989*** (0.532)	9.578*** (0.466)	9.342*** (0.447)	8.915*** (0.572)
Region of residence (ref: Large cities)					
Small and mid-sized cities	0.319 (1.145)	−1.074 (1.807)	1.315 (1.477)	1.040 (1.678)	−0.482 (1.539)
Rural areas	0.197 (1.154)	−0.280 (1.818)	0.602 (1.492)	1.428 (1.620)	−1.287 (1.634)
Living arrangements (ref: Living with more than one person)					
Living with one person	−0.132 (0.310)	0.0412 (0.486)	−0.247 (0.401)	−0.460 (0.433)	0.234 (0.440)
Living alone	−1.049 (1.128)	−3.833** (1.773)	0.831 (1.458)	−1.053 (1.538)	−1.152 (1.655)
Care for a family member with ADL limitations (ref: Does not provide care)					
Provides care	−1.422* (0.771)	0.305 (1.244)	−2.440** (0.979)	−2.454** (0.999)	0.280 (1.221)
Constant	52.73*** (0.949)	52.22*** (1.438)	53.39*** (1.287)	51.34*** (1.493)	54.55*** (1.235)
Observations	22,271	10,351	11,920	12,693	9571
Number of entities	7151	3592	3559	4153	3022

Standard errors in parentheses ***p < .01, **p < .05, *p < .1.

*Note.* 21.1% (1512 out of 7151) of respondents experienced a change in their work status between 2006 and 2020.

The FE panel analysis reveals that changes in individual circumstances are statistically significantly associated with changes in SWB. Transitioning from not working to working is associated with an increase in SWB within individuals in the total sample (β = 1.511, *p* < .01), with a stronger positive association among men (β = 2.947, *p* < .01), while no statistically significant association is observed among women (β = 0.398, *p* > .05). Work status is associated with higher well-being among older adults with both lower and higher education levels, and the association is slightly stronger among those with higher education (β = 1.420, *p* < .01 for lower education; β = 1.596, *p* < .01 for higher education). A decline in self-rated health, from excellent to poor, is associated with a marked decrease in individuals’ SWB, with the greatest declines observed among women (β = −9.710, *p* < .01). Additionally, taking on caregiving responsibilities for a family member with ADL limitations is associated with decreases in SWB among women (β = −2.440, *p* < .05) and among those with lower educational attainment (β = −2.454, *p* < .05), with no statistically significant association observed among men.

Developing three or more ADL limitations is associated with substantial declines in SWB within individuals (β = −7.915, *p* < .01), observed among men (β = −6.091, *p* < .01), women (β = −9.714, *p* < .01), the lower-educated (β = −8.465, *p* < .01), and the higher-educated (β = −6.941, *p* < .01). The frequency of depressive symptoms over the past week shows a dose–response association with changes in SWB. Compared to periods when individuals felt depressed briefly or not at all, experiencing occasional feelings of depression (one or two days) is associated with declines in SWB, for instance, β = −2.077 (*p* < .01) in the total sample, β = −2.175 (*p* < .01) among men, and β = −1.988 (*p* < .01) among women. The declines are more pronounced when feelings of depression occur more frequently (three to four days: β = −3.359, *p* < .01), particularly among men (β = −3.479, *p* < .01). The largest declines are observed among women (β = −5.366, *p* < .01) and the lower-educated (β = −6.098, *p* < .01) when depressive symptoms are persistent (five to seven days).

Improvements in relationship quality with a spouse or children are associated with increases in individuals’ SWB across all groups. For instance, improvement to a high-quality relationship from low-quality relationship with children is associated with increases in SWB in the total sample (β = 9.239, *p* < .01), as well as among men (β = 8.989, *p* < .01), women (β = 9.578, *p* < .01), the lower-educated (β = 9.342, *p* < .01), and the higher-educated (β = 8.915, *p* < .01). Improvements in relationship quality with a spouse are similarly associated with increases in individuals’ SWB across all groups. Transitioning to a high-quality spousal relationship is linked to higher SWB in the total sample (β = 7.646, *p* < .01), as well as among men (β = 6.774, *p* < .01), women (β = 8.114, *p* < .01), the lower-educated (β = 7.150, *p* < .01), and the higher-educated (β = 8.516, *p* < .01).

**Figure 1.**
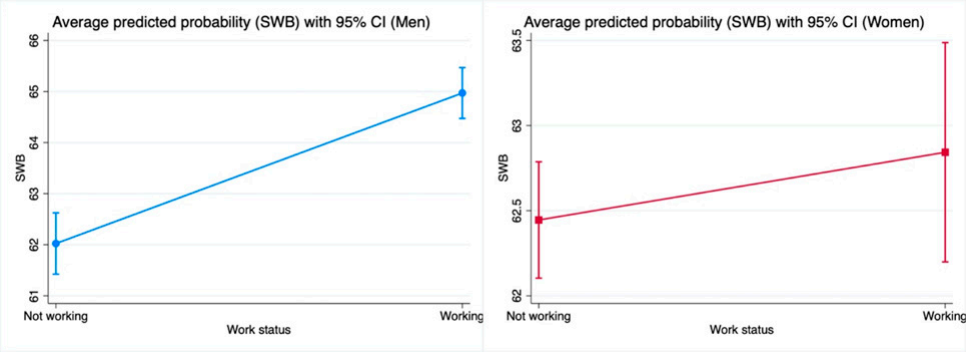
Average predicted probability of well-being with 95% confidence interval, using stratified samples by gender. The values of all other covariates in the model are fixed at their mean values. The figure illustrates the average predicted probability of SWB with a 95% confidence interval, distinguishing between men and women. The *x*-axis represents employment status (not working vs. working), while the *y*-axis denotes SWB. For men, SWB increases from approximately 61.4–62.7 when not working to 64.4–65.5 after switching to working. For women, SWB rises from about 62.1–62.8 when not working to 62.2–63.5 after switching to working.

**Figure 2.**
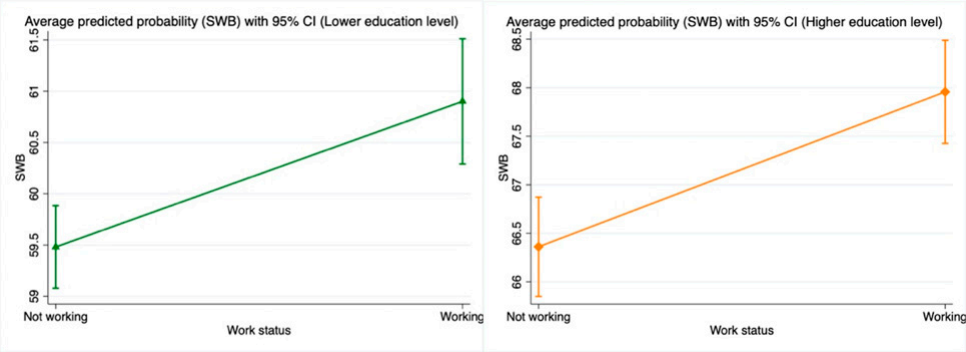
Average predicted probability of well-being with 95% confidence interval, using stratified samples of higher- and lower-education levels. The values of all other covariates in the model are fixed at their mean values. The figure depicts the average predicted probability of SWB with a 95% confidence interval, distinguishing between lower-SES and higher-SES individuals. The *x*-axis represents employment status (not working vs. working), while the *y*-axis denotes SWB. For lower-SES individuals, SWB increases from approximately 59.1–59.9 when not working to 60.3–61.5 after switching to working. For higher-SES individuals, SWB rises from around 59.9–66.9 when not working to 67.4–68.5 after transitioning to working.

Figure 1 presents the FE regression results on the relationship between work status and SWB using stratified samples of men and women, with 95% confidence intervals. Transitioning to work status is associated with higher SWB for men, with relatively narrow confidence intervals, particularly in the “Working” category, indicating more precise estimates. In contrast, for women, transitioning between these work statuses corresponds to a smaller difference in SWB. The flatter slope suggests a weaker association, while the wider confidence intervals, especially in the “Not working” status, indicate greater variability, reflecting the non-significant association for women. Figure 2 presents the predicted probability of SWB by work status for older adults with varying levels of education. Lower and higher education levels show a positive association between work status and SWB, with similar slopes reflecting the transition from not working to working. While individuals with higher education tend to have slightly higher overall SWB, the comparable slope steepness indicates that the relationship between employment status and SWB is consistent across educational levels.

## Discussion

This study examines the relationship between the work status of older people and their SWB, with a particular focus on the moderating roles of gender and educational level. Firstly, a positive statistical association was found between work status and SWB for the overall population, supporting *H1*. This suggests that being employed is generally associated with higher SWB among older people in Korea. This may be partly attributable to the financial pressures that compel many older people to continue working. That is to say that having a job is preferable to being unemployed, as it enables individuals to make a living. However, this interpretation remains tentative, given that factors like financial necessity, job satisfaction, or self-efficacy are not directly examined.

The results also suggest that the association with work status and individual SWB varies by gender. While the positive link between employment status and SWB was stronger for men, no significant association was found for women. Random effects estimates of the association between work status and SWB also show that the association is not statistically significant among older women, as presented in Appendix Table A1. These findings partially support *H2* and indicate that the support for *H1* is primarily driven by men. This implies that men may experience a greater boost in SWB from being employed than women. Thus, the results largely reaffirm the classic theory on the varying effects of work status on SWB depending on gender. As highlighted in the theoretical framework, for men, work identity is known to be stronger than that of women. However, even for men, it is important to note that the association between employment and SWB tends to be modest, especially when viewed alongside much stronger associations with health-related aspects, such as self-rated health, chronic conditions, and ADL limitations, as well as relational aspects involving partners and children. This indicates that work status is less prominently linked to SWB than these other factors. For women, while employment showed no significant association with SWB, providing care to a family member with ADL limitations was significantly negatively associated with their SWB. This pattern was not observed among men, suggesting that the care burden may weigh more heavily on women and could obscure any potential association between employment and SWB.

Korea has traditionally adhered to a male breadwinner model, with a pension system that disproportionately benefits men. Since pension eligibility is largely employment-based, older men receive greater financial security through pensions, while many women face economic insecurity in later life. Among those receiving a pension after contributing to the National Pension Scheme for over 20 years, there were approximately 728,900 men and 120,500 women in 2022, with the number of men being six times that of women (Lee, 2023).

Additionally, lower educational attainment among older women further limits their access to stable, well-paying jobs throughout their lives. Given these structural disadvantages, it is reasonable to expect that employment status is linked to higher SWB of older women, as it provides greater financial security compared to not working. However, other factors may be more influential since this association was not statistically significant.

One possible explanation is involuntary employment. In Korea, older women may struggle to secure adequate jobs, making their decision to work less about self-efficacy or fulfilment and more about economic necessity or a lack of alternatives. Involuntary employment is known to have no positive association with well-being (Sohier, 2019), which may explain why work does not significantly enhance SWB for women. This distinction, however, is complicated to capture in the Korean context: while financial motives may drive older adults to remain in work, this does not necessarily imply that their employment is involuntary, as work can simultaneously fulfil economic, social, and psychological needs. Further research using more nuanced measures of work motivation would help clarify these dynamics.

Another factor could be the nature of employment available to older women. Career breaks due to caregiving responsibilities and gendered occupational patterns often push them into precarious, low-paying jobs. Many older women work in the service sector, which is characterised by lower wages and less favourable working conditions (Kim & Gordon, 2014). Kim and his colleagues (2018) highlight the increasing *feminisation of precariousness* in Korea, which may stem from the high prevalence of women in service occupations where poor job quality is the norm. Empirical research indicates that the relationship between work and well-being is highly gendered, largely due to disparities in financial returns (Baslevent & Kırmanoğlu, 2017). Women frequently encounter lower wages, fewer career advancement opportunities, and higher rates of precarious employment, particularly in sectors such as caregiving, hospitality, and retail. These factors may contribute to the weaker association between work status and SWB among older women in Korea.

The observed result also supports the expectation (H3) that older adults with higher education levels report greater well-being when working, in line with cumulative disadvantage and resource theories of aging. However, the difference in the strength of the association was modest (β = 1.420, *p* < .01 for lower education; β = 1.596, *p* < .01 for higher education). This may be attributed to the prevailing circumstances in the Korean context, where participation in employment may have favorable consequences for individuals’ SWB due to financial necessities, regardless of educational level. For instance, Chou et al. (2004) found that older Hong Kong Chinese with work-related income experience greater SWB than those relying solely on government assistance, as employment understandably alleviates financial strain. Alternatively, the mechanisms linking employment to SWB may differ by education level: self-efficacy or confidence may play a greater role for those with higher levels of education, while financial stability may be more relevant for those with lower levels of education.

Another possibility is that the employment opportunities available to older adults are relatively homogeneous, which limits variation in their effects across different educational levels. In other words, highly and less-educated older adults may end up in similar types of secondary employment in later life, which may reduce differences in outcomes across educational groups, though this would require further evidence to confirm.

Overall, the findings suggest that activity theory may not be universally applicable to all older individuals and thus requires careful consideration in policy development. As demonstrated in the results, health-related indicators and relational and emotional support systems strongly correlate with older adults’ SWB than work status. Policy initiatives should therefore adopt a more nuanced approach, prioritising health and emotional support alongside opportunities for engagement in the labour market, rather than assuming that work-related activity alone is sufficient to sustain well-being in later life. In particular, for older women, policies could prioritize support for health and caregiving responsibilities, address the disproportionate caregiving burden they may face, and effectively promote SWB alongside employment.

## Limitations and Future Directions

As with any research, this study is not without limitations, particularly in its empirical approach and theoretical assumptions. Fixed effects models are well-suited to this analysis, as they control for time-invariant and unobserved individual characteristics, such as personality traits, that account for a substantial share of the variation in subjective well-being. Furthermore, the Hausman test of endogeneity indicates that the endogeneity bias in the FE estimation is not significant. Nevertheless, estimation results may still be biased if time-varying omitted variables closely associated with both employment status and SWB are present. Also, FE models do not entirely eliminate concerns about reverse causality. For instance, older individuals with higher SWB who enjoy their jobs may be more likely to stay in work, while those with lower SWB may exit. While this highlights a limitation of the analytical model, a policy implication can still be drawn from this. For instance, measures to enhance the well-being of older adults, such as mental health support, social engagement opportunities, or improved job quality, may help sustain employment in later life. This suggests that policies may need to go beyond financial incentives and address the broader conditions that support well-being.

It should also be noted that this study assumes, but does not directly test, the mechanisms through which employment influences SWB, particularly across gender and educational levels. While financial necessity, self-efficacy, and job quality are plausible explanations, they cannot be empirically verified within the current framework. Acknowledging that binary work status may obscure important aspects of employment conditions, supplementary analyses incorporating qualitative dimensions of employment are presented in Appendix Table A2. Future qualitative research could explore how older adults perceive the role of work status regarding their SWB, shedding light on the subjective meanings and motivations behind employment. Additionally, survey measures capturing job quality, voluntary versus involuntary employment, and perceptions of financial security could provide a more nuanced understanding of these complex dynamics. As mentioned, in Korea, many retire from their primary job in midlife but re-enter the workforce and continue working in later life. Retirement is often a temporary transition rather than a permanent exit from the labour market. As a result, the “not working” category broadly includes both retired and unemployed individuals, and the data do not specifically capture transitions between retirement and employment. Although previous research suggests that unemployment history is relevant to SWB (Böckerman & Ilmakunnas, 2009), it is impossible to reliably isolate and examine the effects of unemployment history within the scope of this study.

This study provides a more nuanced understanding of later-life employment across gender and educational groups. The findings underscore the importance of considering gendered aspects of work and well-being, aligning with theoretical perspectives that emphasize the diverse meanings and impacts of employment on men and women. Understanding the variations in the experiences of older people regarding work status and well-being is crucial for developing targeted interventions and policies that enhance their overall well-being. The case of Korea is valuable for understanding its specific context and contributes to the global discourse on aging, work, and well-being, particularly in countries where longer working lives have become or will become a necessity. By exploring how gender and education shape the relationship between work and well-being, this study offers insights that extend beyond Korea, providing a basis for policies and practices in other societies grappling with similar challenges.

Given high poverty and suicide rates among older adults in Korea, policies should strengthen support systems and improve job quality to enhance well-being. For older women, it is important to ensure that employment enhances well-being through age- and gender-inclusive work environments, flexible scheduling, protection against discrimination, access to mental health services, and opportunities for social engagement and meaningful work. While institutional contexts vary, the relationship between later-life employment and SWB is an increasingly important policy concern across aging societies. Accordingly, the findings have broader relevance beyond South Korea, as many high-income countries face comparable pressures, such as welfare retrenchment, inadequate pension coverage, and gendered disparities in pensions and income (Ebbinghaus, 2021; Foster, 2023), that contribute to financial insecurity in later life.

## Supplemental Material

Supplemental Material - Gender Differences in Work and Well-Being in Later Life Supplemental Material for Gender Differences in Work and Well-Being in Later Life by Juryung Kaitlyn Cho in Research on Aging
