# Supplementary material for: Gender Differences in Work and Well-Being in Later Life
Source: Res Aging. 2025 Jun 21;48(1):44–55. doi: 10.1177/01640275251353217 (PMC12559366; doi:10.1177/01640275251353217)
Supplement: Supplemental Material - Gender Differences in Work and Well-Being in Later Life [file sj-pdf-1-roa-10.1177_01640275251353217.pdf]

Appendix

Table A1. Regression of SWB on work status

Model	(1)	(2)	(3)	(4)	(5)
	SWB	SWB	SWB	SWB	SWB
Sample	Total	Men	Women	Lower edu (Middle school or lower)	Higher edu (High school or higher)
Estimation model	Random effects				
**Work status (ref: Not working)**					
Working	0.998***	2.229***	0.0501	1.176***	0.325
	(0.232)	(0.349)	(0.322)	(0.324)	(0.328)
**Self-rated health status (ref: Excellent)**					
Very good	-2.443***	-1.911**	-3.117***	-2.177**	-2.453***
	(0.598)	(0.804)	(0.902)	(1.021)	(0.711)
Good	-4.836***	-4.234***	-5.550***	-4.644***	-4.327***
	(0.602)	(0.817)	(0.903)	(1.015)	(0.729)
Fair	-9.132***	-8.489***	-9.828***	-8.826***	-7.591***
	(0.631)	(0.873)	(0.931)	(1.034)	(0.826)
Poor	-14.67***	-14.82***	-14.75***	-14.35***	-13.11***
	(0.811)	(1.179)	(1.144)	(1.194)	(1.326)
**ADL limitations (ref:0)**					
1	-2.663**	-3.229**	-1.907	-1.325	-6.198***
	(1.146)	(1.633)	(1.616)	(1.344)	(2.305)
2	-6.872***	-6.716***	-6.216**	-6.629***	-7.842**
	(1.848)	(2.339)	(3.080)	(2.252)	(3.300)
3+	-9.851***	-8.972***	-10.40***	-8.885***	-13.25***
	(0.920)	(1.262)	(1.389)	(1.100)	(1.743)
**Depressed mood over the past week (ref: Felt that way briefly or did not feel that way at all)**					
Sometimes felt that way (for a day or two)	-2.868***	-3.082***	-2.695***	-2.570***	-3.095***
	(0.229)	(0.360)	(0.297)	(0.301)	(0.354)
Often felt that way (for about 3 to 4 days)	-4.978***	-4.819***	-5.079***	-4.713***	-4.938***
	(0.423)	(0.683)	(0.537)	(0.512)	(0.782)
Always felt that way (for about 5 to 7 days)	-7.060***	-5.731***	-8.026***	-7.557***	-6.097***
	(0.799)	(1.261)	(1.029)	(0.989)	(1.378)
**Marital status (ref: Married)**					
Not married	-9.351	-13.31	-5.664	-4.817	-13.12
	(9.576)	(13.59)	(13.48)	(14.22)	(12.64)
**Relationship quality with spouse (ref: low)**					
High	9.390***	8.442***	10.04***	8.741***	10.20***
	(0.266)	(0.438)	(0.334)	(0.337)	(0.436)
**Relationship quality with children (ref: low)**					
High	11.22***	11.28***	11.26***	11.49***	10.37***
	(0.303)	(0.456)	(0.408)	(0.382)	(0.500)
**Region of residence (ref: large cities)**					
Small and mid-sized cities	2.166***	2.386***	1.971***	2.479***	1.781***
	(0.300)	(0.447)	(0.401)	(0.431)	(0.404)

Rural areas	0.777**	0.887*	0.744*	1.694***	1.084*
	(0.333)	(0.494)	(0.447)	(0.429)	(0.560)
**Living arrangements (ref: Living with more than one person)**					
Living with one person	-0.00336	0.301	-0.196	-0.261	0.851***
	(0.221)	(0.336)	(0.293)	(0.305)	(0.319)
Living alone	-0.593	-2.417	0.721	-0.405	-0.848
	(0.976)	(1.499)	(1.283)	(1.354)	(1.385)
**Care for a family member with ADL limitations (ref: no)**					
Yes	-2.010***	-0.263	-3.161***	-2.767***	-0.328
	(0.644)	(1.031)	(0.821)	(0.825)	(1.035)
Constant	50.79***	49.81***	51.61***	49.17***	52.33***
	(0.676)	(0.947)	(0.987)	(1.086)	(0.885)
Observations	22,271	10,351	11,920	12,693	9,571
Number of entities	7,151	3,592	3,559	4,153	3,022

Standard errors in parentheses
*** p<0.01, ** p<0.05, * p<0.1

Table A2. Regression of life satisfaction on employment characteristics

	Model 1	Model 2	Model 3	Model 4	Model 5	Model 6	Model 7	Model 8	Model 9	Model 10
Sample	Total	Men	Women	Low-edu	High-edu	Total	Men	Women	Low-edu	High-edu
Estimation model	Fixed effects					Random effects				
**Job security (ref: very secure)**										
Fairly secure	-1.618** (0.803)	-1.887** (0.957)	-0.645 (1.486)	-1.287 (1.259)	-1.821* (1.040)	-2.590*** (0.623)	-2.439*** (0.731)	-2.657** (1.196)	-2.031** (0.980)	-2.982*** (0.799)
Fairly insecure	-3.264*** (0.865)	-3.686*** (1.041)	-2.010 (1.571)	-2.594* (1.331)	-3.825*** (1.139)	-5.368*** (0.656)	-5.478*** (0.777)	-4.937*** (1.235)	-4.228*** (1.015)	-6.132*** (0.856)
Very insecure	-3.521*** (1.128)	-4.449*** (1.378)	-1.300 (1.980)	-3.398** (1.647)	-3.358** (1.583)	-7.557*** (0.844)	-8.339*** (1.016)	-5.842*** (1.532)	-6.756*** (1.229)	-7.533*** (1.199)
**Weekly working days (ref: 1 day)**										
Working days (unit: 1 day)	0.347* (0.178)	0.525** (0.225)	0.0144 (0.293)	0.386 (0.235)	0.289 (0.278)	0.178 (0.125)	0.417*** (0.160)	-0.206 (0.201)	0.377** (0.166)	-0.166 (0.192)
**Weekly working hours (ref: 1 hour)**										
Working hours (unit: 1 hour)	0.0122 (0.0109)	0.0399*** (0.0142)	-0.0294* (0.0170)	0.0105 (0.0151)	0.0145 (0.0159)	-0.00247 (0.00818)	0.0216** (0.0108)	-0.0416*** (0.0126)	0.00383 (0.0113)	-0.0127 (0.0118)
**Employment status (ref: part-time)**										
Full-time	1.165 (1.784)	0.888 (3.064)	1.197 (2.217)	-0.232 (3.062)	1.745 (2.240)	1.660** (0.697)	2.099* (1.133)	0.769 (0.930)	1.237 (0.990)	1.652* (0.978)
**Job types (ref: Professional and clerical occupations)**										
Service and sales occupations	-1.202 (1.558)	-1.542 (2.037)	-0.839 (2.518)	-2.711 (6.146)	-1.306 (1.615)	-3.316*** (0.571)	-2.369*** (0.724)	-4.731*** (1.027)	-4.606*** (1.566)	-2.345*** (0.636)
Manual and elementary occupations	-2.735 (1.675)	-3.076 (2.073)	-1.841 (2.897)	-5.132 (6.131)	-2.293 (1.820)	-3.920*** (0.551)	-3.647*** (0.648)	-4.700*** (1.055)	-3.914*** (1.514)	-3.535*** (0.651)

Standard errors in parentheses
*** p<0.01, ** p<0.05, * p<0.1

Note: All models include the same covariates as those in Table 2.

To better capture the quality of employment beyond binary work status, several indicators of employment characteristics were incorporated: perceived job security, weekly working days and hours, employment status (full-time vs. part-time), and job types. Models 1 to 5 present fixed effects (FE) estimates, while Models 6 to 10 present random effects (RE) estimates. Model 1 and Model 6 show that lower job security is statistically significantly associated with lower SWB. As shown in Model 2 and Model 7, negative associations between job insecurity and SWB are more pronounced among men compared to women. While lower job security is significantly associated with lower SWB among women in Model 8, this association is not statistically significant in Model 3. Positive associations between an increase in working days and SWB are observed among men (Model 2 and Model 7). An increase in working hours is negatively associated with women's SWB (Model 3 and Model 8), whereas it is positively associated with men's SWB (Model 2 and Model 7). Full-time employment is associated with higher SWB compared to part-time employment, particularly among men (Model 7) and higher-educated individuals (Model 10). Regarding job types, service and sales occupations, as well as manual occupations, are statistically significantly associated with lower SWB compared to professional and clerical occupations, as RE estimates indicate.
